# Metabolism-related MOGS Gene is Dysregulated After Peripheral Nerve Injury and Negatively Regulates Schwann Cell Plasticity

**DOI:** 10.1007/s12031-022-02024-8

**Published:** 2022-05-16

**Authors:** Yunsong Zhang, Miao Yang, Yinying Shen, Sheng Yi, Xinghui Wang

**Affiliations:** 1 Key Laboratory of Neuroregeneration of Jiangsu and Ministry of Education, Co-Innovation Center of Neuroregeneration, NMPA Key Laboratory for Research and Evaluation of Tissue Engineering Technology Products, Nantong University, Nantong, 226001 Jiangsu China; 2 Department of Pharmacy, Yancheng City No. 1 Peoples’ Hospital, Yancheng, 224000 Jiangsu China

**Keywords:** Peripheral nerve regeneration, Metabolism, Schwann cell, MOGS

## Abstract

Cellular metabolism is essentially linked to tissue remodeling and organ regeneration. *MOGS*, a gene that encodes cellular metabolism-related protein mannosyl-oligosaccharide glucosidase, was found to be upregulated in nerve segments after peripheral nerve injury. Bioinformatic analyses identified upstream regulators of MOGS and MOGS-associated genes and indicated the significant involvement of cellular metabolism in peripheral nerve regeneration. Functional assessment showed that siRNA-mediated knockdown of MOGS led to elevated proliferation, migration, and differentiation of Schwann cells, indicating the negative regulation of MOGS on Schwann cell plasticity. Schwann cells transfected with MOGS siRNA also showed lower expression of fatty acid synthase (FASN), demonstrating that dysregulated MOGS in Schwann cells may affect neuronal behavior through the metabolic coupling between Schwann cells and axons. Taken together, this study demonstrated that MOGS may be a key regulating factor of Schwann cells and neuronal phenotype during peripheral nerve regeneration.

**Supplementary Information:**

The online version contains supplementary material available at 10.1007/s12031-022-02024-8.

## Introduction

Peripheral nerve regeneration is a well-orchestrated process involving a series of cellular events, including the reprogramming of glial cells, infiltration of cytotoxic natural killer cells, activation of macrophages, formation of endothelial tubules, and elongation of axons (Cattin et al. 2015; Chandran et al. 2016; Davies et al. 2019; Jessen and Mirsky 2016). Cellular metabolism is largely involved in these biological activities as tissue repair and wound healing are highly energy-consuming processes (Magadum and Engel 2018; Shyh-Chang et al. 2013). For instance, major metabolic regulators mTOR and Akt kinases are critical factors for axon regeneration (Mahar and Cavalli 2018; Park et al. 2008; Terenzio et al. 2018). Elevated macrophage metabolism stimulates macrophage activation and facilitates peripheral nerve regeneration (Jha et al. 2021; Langston et al. 2017). Altered metabolism of Schwann cells, the unique glial cells in peripheral nerves, influences peripheral nerve myelination and maturation (Jha et al. 2020). Moreover, Schwann cells also regulate the fate of injured axons by manipulating glycolytic activities and contributing to the maintenance of axonal integrity via axon-glia metabolic coupling (Babetto et al. 2020; Beirowski et al. 2014).

Besides glycolytic metabolism, lipid metabolism in glial cells also plays important roles in axon growth and peripheral nerve regeneration. Following peripheral nerve injury, the expressions of many lipid metabolism-related genes were found to be upregulated in satellite glial cells, which are peripheral glial cells localized in dorsal root ganglia (DRG). Conditional deletion of FASN in satellite glial cells impairs neurite outgrowth and hinders axon elongation (Avraham et al. 2020). Activation of lipid metabolism-associated canonical signaling pathways and upregulation of metabolism-related genes were also observed in the injured peripheral nerve segments (Yi et al. 2015). Considering the large population of Schwann cells in peripheral nerves, the expressions of these metabolism-related genes may be augmented in Schwann cells, and these differentially expressed genes may mediate phenotype modulation of Schwann cells.

MOGS, also named GCS1, encodes mannosyl-oligosaccharide glucosidase, a protein located in the lumen of the endoplasmic reticulum (ER) that catalyzes N-linked oligosaccharide processing (Sadat et al. 2014). MOGS has also been reported to alleviate ER stress and inhibit the elevation of ER stress-associated lipogenesis genes, such as *FASN*, indicating its critical role in lipid metabolism (Liu et al. 2018). Given the significant involvement of MOGS in cellular metabolism and the potential participation of MOGS in peripheral nerve regeneration, this study examined the expression changes of MOGS after peripheral nerve injury using a rat sciatic nerve crush model and investigated the biological functions of MOGS in Schwann cells.

## Materials and Methods

### Animal Surgery

Animal surgery was performed according to the Institutional Animal Care Guidelines of Nantong University. This study received ethics approval by the Administration Committee of Experimental Animals, Nantong University, Jiangsu Province, China (approval No. 20170302–017) on March 2, 2017. Adult, male Sprague–Dawley (SD) rats were purchased from Nantong University and subjected to nerve crush injury as previously described (Yi et al. 2015). Briefly, after anesthetization, rat sciatic nerve was exposed and crushed with a forceps at 10 mm above the bifurcation into the tibial and common fibular nerves. Rats were randomly divided into five groups with three rats in each group. Sciatic nerves at the injured site were collected at 1 day, 4 days, 7 days, and 14 days after nerve injury. Uninjured sciatic nerves were collected and designated as 0 day control.

### Schwann Cell Isolation, Culture, and Transfection

Neonatal SD rats were purchased from Nantong University, and their sciatic nerves were extracted for cell collection. Cells were treated with anti-Thy1.1 (1:1000, M7898, Sigma, St. Louis, MO, USA) and rabbit complement (Invitrogen, Carlsbad, CA, USA) to remove fibroblasts. Purified Schwann cells were cultured in DMEM (10–013-CVR, Corning, NY, USA) containing 10% FBS (10099141c, Gibco, Grand Island, NY, USA), 1% penicillin and streptomycin (c0222, Beyotime, Shanghai, China), 2 μM forskolin (Sigma), and 10 ng/ml HRG (R&D Systems Inc., Minneapolis, MN, USA). Cultured Schwann cells were transfected with siRNAs against MOGS (siRNA-1: RibiBio, siG2010140505022069, GUCUAUUUCGGCAUGAAGA, siRNA-2: RibiBio, siG2010140505023161, UCGGCAACAUAUCUAUGAU, and siRNA-3: RibiBio, siG2010140505024253, GUAAAGAGCCACCUAAACA) or a random sequence non-targeting negative control (RibiBio, Guangzhou, Guangdong, China) using Lipofectamine RNAiMAX transfection reagent (Invitrogen).

### Cell Differentiation

Cellular differentiation was mediated by culturing cells in a differentiation medium group containing DMEM/F12 (10–092-CVR, Corning), 0.5% FBS (Gibco), 1% penicillin and streptomycin (Beyotime), 20 ng/ml HRG (R&D Systems Inc.), and 1 mM db-cAMP (Sigma). Cells in the control group were cultured in DMEM/F12 containing 0.5% FBS and 1% penicillin and streptomycin. Media were refreshed every day, and cells were cultured for 3 days prior to real-time RT-PCR experiments.

### RT-PCR

Total RNA was extracted from rat sciatic nerve tissues and Schwann cells using TRIzol reagent (Invitrogen). RNA was reverse-transcribed, and RT-PCR was conducted using SYBR Green Premix Ex Taq (TaKaRa, Dalian, Liaoning, China) on a StepOne Real-time PCR System (Applied Biosystems, Foster City, CA, USA). Gene expressions were calculated using the comparative 2^–ΔΔCt^ method, with GAPDH as the internal control. Primer sequences were MOGS, 5′-CTTCTGCCACCAACCACTCC-3′ (forward) and 5′-CCAGGCAAGCCAAGGTATCG-3′ (reverse); P0, 5′-CGTGATCGGTGGCATCCTC-3′ (forward) and 5′-GGCATACAGCACTGGCGTCT-3′ (reverse); p75, 5′-CTGCTGATTCTAGGGATGTCCT-3′ (forward) and 5′-ATGTAACACTGTCCAGGCAGG-3′ (reverse); GAPDH, 5′-ACAGCAACAGGGTGGTGGAC-3′ (forward) and 5′-TTTGAGGGTGCAGCGAACTT-3′ (reverse); FASN, 5′-GCTTGGTGAACTGTCTCCGA-3’ (forward) and 5′-GTGAGATGTGCTGCTGAGGT-3′ (reverse).

### Bioinformatic Analysis

TargetScan (http://www.targetscan.org/vert_72/), miRWalk (http://mirwalk.umm.uni-heidelberg.de/), miRcode (http://mircode.org/), and RNA22 v2 (https://cm.jefferson.edu/rna22/Interactive/) were applied to predict potential upstream miRNAs of MOGS. Venn online tool (Venny 2.1.0; http://bioinfogp.cnb.csic.es/tools/venny/index.html) was utilized to screen overlapping predicted upstream miRNAs. Cytoscape software v3.8.0 (https://cytoscape.org/) was utilized to identify MOGS-associated genes as well as involved Gene Ontology (GO) and Kyoto Enrichment of Genes and Genomes (KEGG) categories (Shannon et al. 2003).

### EdU Incorporation Assay

Schwann cells transfected with siRNA against MOGS or a negative control were seeded onto 96-well plates pre-coated with poly-l-lysine. Cell proliferation was observed using the Cell-Light EdU DNA cell proliferation kit (Ribobio) according to the manufacturer’s instructions. Briefly, after treatment with 50 μM EdU for 24 h, cells were fixed with 4% paraformaldehyde in PBS for 30 min and stained with Apollo 567 fluorescent dyes and Hoechst 33,342. Images were taken under a Leica Model DMi8 microscope (Leica Microsystems CMS GmbH, Bensheim, Germany).

### Immunostaining

Schwann cells transfected with siRNA against MOGS or a negative control were fixed with 4% paraformaldehyde in PBS, blocked with Immunol Staining Blocking Buffer (Beyotime, Shanghai, China) for 30 min, incubated with primary rabbit anti-Ki67 antibody (1:200, ab16667, Abcam, Cambridge, MA, USA) overnight at 4 ℃, followed by Cy3-conjugated Affinipure Goat Anti-Rabbit IgG secondary antibody (1:400, SA00009-2, ProteinTech Group, Chicago, IL, USA) for 2 h at room temperature. Nuclei were stained with DAPI (0100–20, SouthernBiotech, Birmingham, AL, USA) for 10 min at 25 ℃. Images were taken with a ZEISS Imager M2 (Carl Zeiss Microscopy GmbH, Jena, Germany).

### Transwell Migration Assay

Schwann cells transfected with siRNA against MOGS or a negative control were resuspended in 100 μL DMEM (without FBS) and seeded onto the upper chamber of a 6.5-mm Transwell with 8-μm pores (Costar, Cambridge, MA, USA). The bottom chamber of the Transwell was filled with 500 μL 10% FBS-containing complete culture medium. Schwann cells were cultured for 24 h and then the upper surface of the upper chamber was cleaned with a cotton swab. The bottom surface of the upper chamber was stained with 0.1% crystal violet to label migrated cells. Images were taken with a Leica Model DMI3000B microscope (Leica Microsystems).

### Wound Healing Assay

Schwann cells transfected with siRNA against MOGS or a negative control were seeded onto 6-well plates with wound healing culture inserts (ibidi, Martinsried, Germany) placed in the middle of the culture plates. After cell confluence, wound healing culture inserts were removed, and Schwann cells were cultured for additional 9 h. Images were taken with a Leica Model DMI3000B microscope (Leica Microsystems). Image-Pro Plus (Media Cybernetics, Silver Springs, MD, USA) was used to determine the surface area of clear space.

### Live Cell Imaging

Schwann cells transfected with siRNA against MOGS or a negative control were cultured in a Nunc™ Lab-Tek™ chamber (ThermoFisher Scientific, Waltham, MA, USA). Time-lapse images were taken with an Olympus IX81 microscope (Olympus, Tokyo, Japan). Images were taken every 5 min during the 12 h of observation. Xcellence Sequence (Olympus) and ImageJ (National Institutes of Health, Bethesda, MD, USA) were used to determine cell migration distance and velocity, respectively.

### Quantification and Statistical Analysis

Statistical analysis was performed using GraphPad Prism 6.0 (GraphPad Software, Inc., La Jolla, CA, USA) software. No data were excluded from the analyses. Data were summarized from at least three replicates and shown as mean ± SEM in the figure legends. D’Agostinl-Pearson omnibus normality test and Brown-Forsythe test were performed first before parametric or non-parametric tests. Differences in means were analyzed by Student’s *t* test or one-way analysis of variance (ANOVA) and Tukey’s honestly significant difference (HSD) post hoc test when the normality of data distribution and equal variance between groups was met; otherwise, non-parametric tests (Wilcoxon) were applied. Student’s *t* test was used to analyze functional differences between Schwann cells after MOGS knockdown. The one-way ANOVA was used to analyze the mRNA expression of MOGS in nerve stumps at different time points after sciatic nerve injury. A *p*-value < 0.05 was considered significant (*p < 0.05).

## Results

### MOGS Gene Expression was Elevated in the Injured Nerve Segments Following Peripheral Nerve Injury

**Fig. 1:**
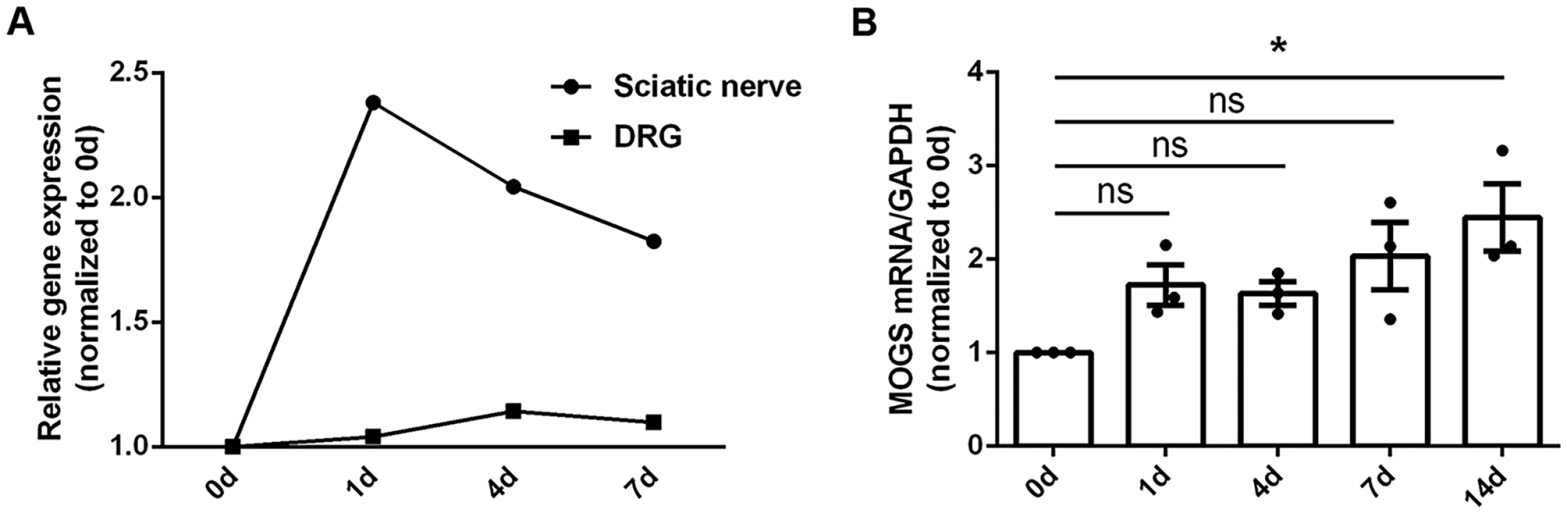
Dynamic expression patterns of MOGS. **A** Sequencing analysis of the relative temporal expression patterns of MOGS in sciatic nerves and DRG at 0, 1, 4, and 7 days after sciatic nerve injury. **B** RT-PCR quantification of the relative expression levels of MOGS in sciatic nerves at 0, 1, 4, 7, and 14 days after sciatic nerve injury. *p-value < 0.05 versus the control (day 0) group (*n* = 3; mean ± SEM; one-way ANOVA). d day(s)

The relative mRNA abundance of MOGS in rat sciatic nerves and DRG at multiple time points following sciatic nerve injury were evaluated using obtained sequencing data from NCBI database PRJNA394957 (SRP113121) (Zhao and Yi 2019) and PRJNA547681 (SRP200823) (Shen et al. 2020). Sequencing data indicated that the gene expression of MOGS in DRG was generally unchanged (Fig. 1A). However, in sciatic nerves, the gene expression of MOGS robustly increased by about 2.4-fold compared to its expression in the uninjured 0 day control at 1 day after nerve injury. Substaining elevated MOGS expression was detected at later time points (Fig. 1A). Changes in MOGS expression in sciatic nerves were further validated by RT-PCR experiments, which were consistent with sequencing data, and revealed persistent upregulation of MOGS, implying the potential regulatory effects of MOGS on Schwann cell behavior (Fig. 1B).

### Construction of MOGS-centered Genetic Network

**Fig. 2:**
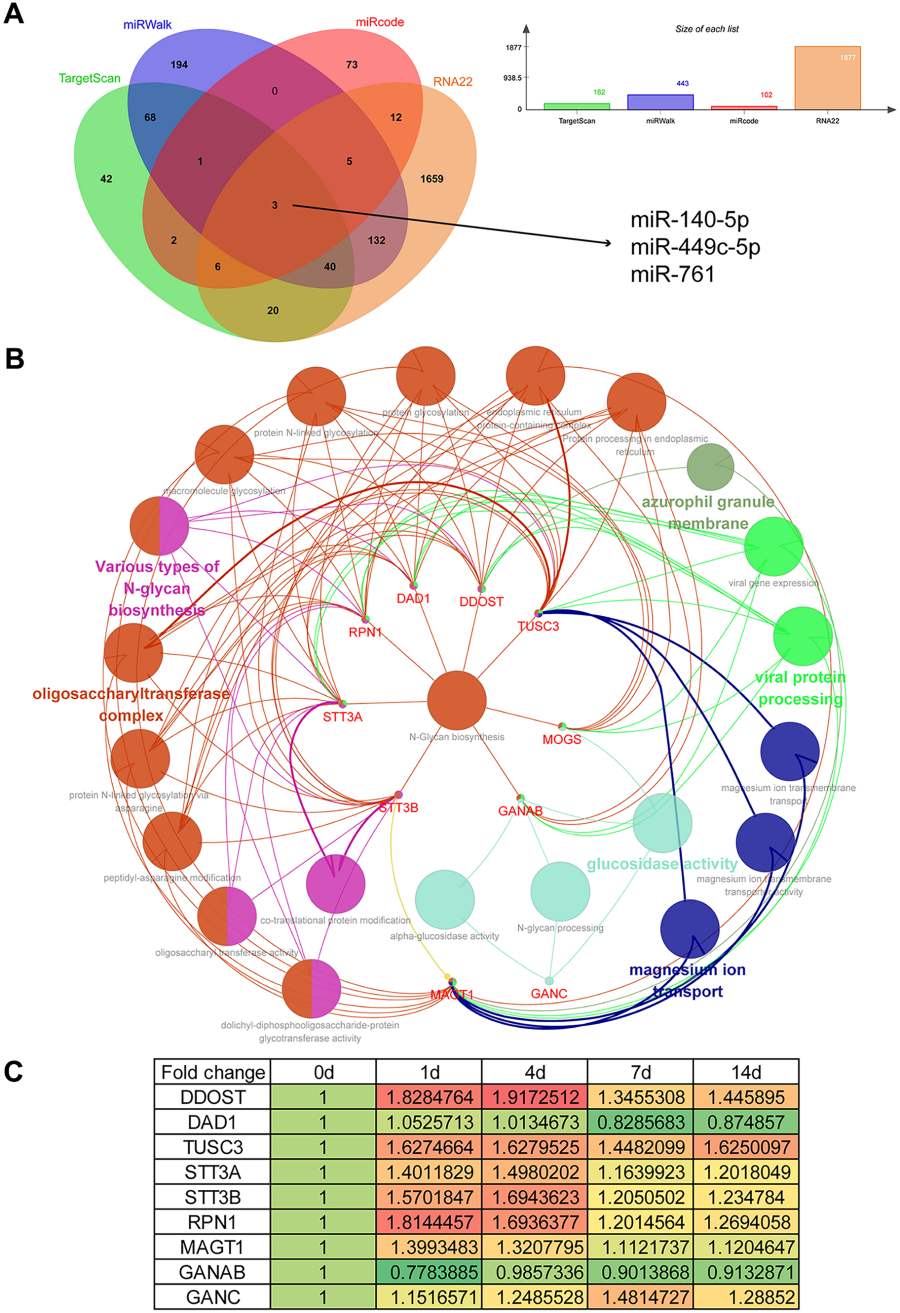
Bioinformatic analysis of MOGS-related genes. **A** Venn diagram of upstream miRNAs of MOGS predicted by TargetScan, miRWalk, miRcode, and RNA22. **B** The genetic network of MOGS-associated genes and GO/KEGG categories. Different colors of network nodes represent different GO/KEGG categories, with orange red representing oligosaccharyltransferase complex, pink representing various types of N-glycan biosynthesis, dark blue representing magnesium ion transport, light blue representing glucosidase activity, light green representing viral protein processing, and dark green representing azurophile granule membrane. **C** The relative expression levels of genes in the MOGS-centered network. Red represents upregulation, while green represents downregulation

Bioinformatic tools were applied to determine upstream regulators and downstream factors of MOGS. The miRNA-mRNA prediction software TargetScan, miRWalk, miRcode, and RNA22 screened 182, 443, 102, and 1877 miRNAs as candidate upstream regulatory miRNAs of MOGS, respectively. Joint analyses of these prediction software indicated that miR-140-5p, miR-449c-5p, and miR-761 might be upstream regulatory miRNAs of MOGS (Fig. 2A).

Besides upstream regulators, target genes of MOGS were also identified using Cytoscape software. A total of 14 genes, including dolichyl-diphosphooligosaccharide protein glycosyltransferase non-catalytic subunit (*DDOST*), defender against cell death 1 (*DAD1*), tumor suppressor candidate 3 (*TUSC3*), isochorismatase domain containing 1 (*ISOC1*), isochorismatase domain containing 2 (*ISOC2*), leucine-rich repeat kinase 2 (*LRRK2*), Y-Box binding protein 1 (*YBX1*), translocase of inner mitochondrial membrane 44 (*TIMM44*), STT3 oligosaccharyltransferase complex catalytic subunit A (*STT3A*), STT3 oligosaccharyltransferase complex catalytic subunit B (*STT3B*), ribophorin I (*RPN1*), magnesium transporter 1 (*MAGT1*), glucosidase II alpha subunit (*GANAB*), and glucosidase alpha neutral C (*GANC*) were identified to be associated with MOGS. Among these 14 MOGS-associated genes, nine genes, i.e., *DDOST*, *DAD1*, *TUSC3*, *STT3A*, *STT3B*, *RPN1*, *MAGT1*, *GANAB*, and *GANC*, were linked with GO terms and KEGG pathways (Fig. 2B). Most MOGS-associated genes were involved in metabolic processes, including oligosaccharyltransferase complex, *N*-glycan biosynthesis, and glucosidase activity.

The expression profiles of genes involved in the MOGS-centered network were determined from the NCBI database PRJNA394957 (SRP113121), and the temporal changes of genes were displayed in a heatmap (Fig. 2C). Consistent with the expression changes of *MOGS*, the expressions of many metabolism-associated genes, including *DDOST*, *TUSC3*, *STT3A*, *STT3B*, *RPN1*, *MAGT1*, and *GANC*, were elevated after peripheral nerve injury.

### MOGS-modulated Schwann Cell Differentiation

**Fig. 3:**
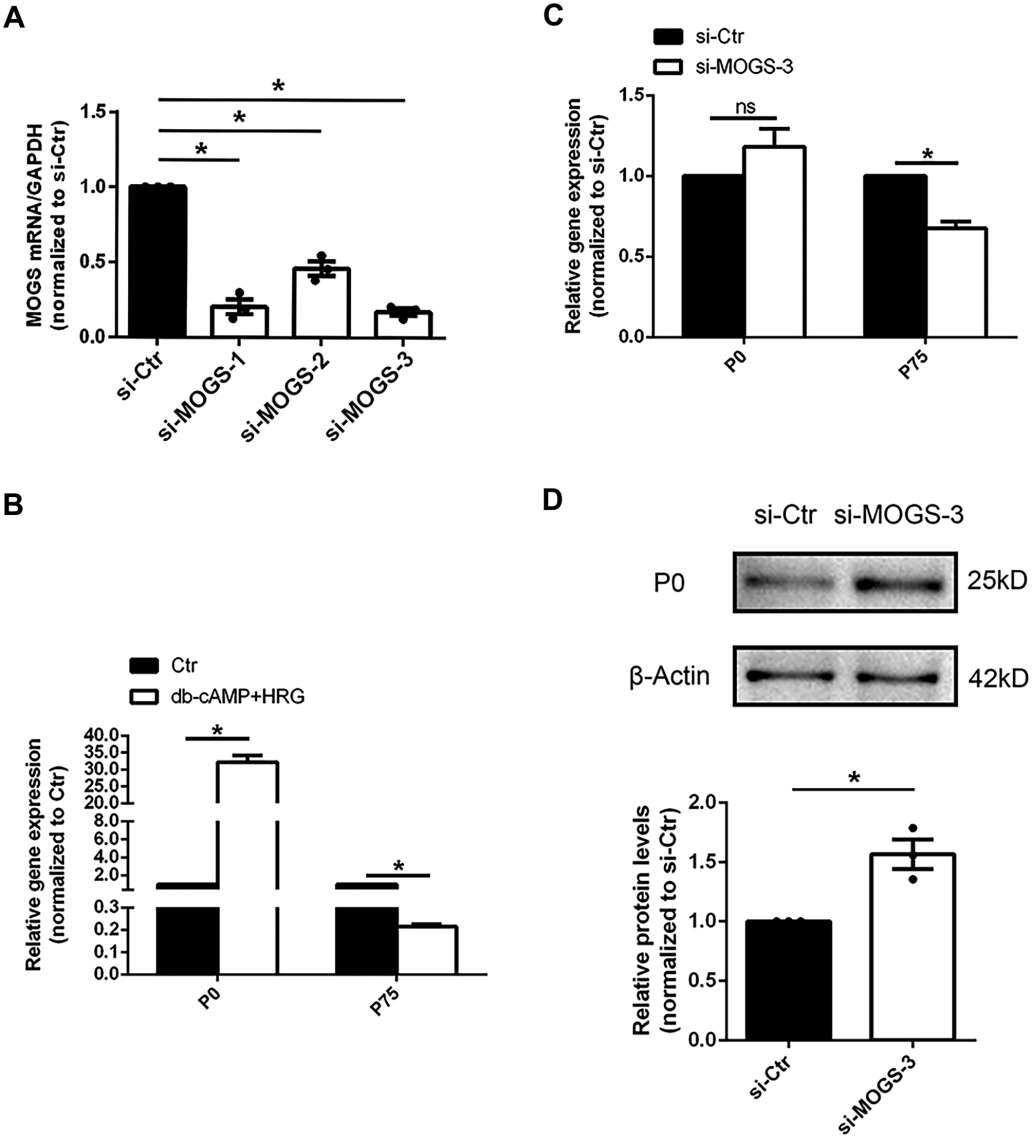
MOGS affects Schwann cell differentiation. **A** Transfection of Schwann cells with si-MOGS decreased the mRNA abundance of MOGS. *p-value < 0.05 versus si-Ctr (*n* = 3, mean ± SEM; Student’s *t*-test). **B** The mRNA levels of P0 and p75 in Schwann cells cultured in differentiation culture medium and control medium, respectively. *p-value < 0.05 versus Ctr (*n* = 3, mean ± SEM; Student’s *t*-test). Ctr control medium. **C** The mRNA levels of p75 were higher in siRNA control (si-Ctr)-transfected Schwann cells cultured in differentiation culture medium than siRNA-MOGS-transfected Schwann cells (si-MOGS-3) cultured in differentiation culture medium. *p-value < 0.05 versus si-Ctr (*n* = 3, mean ± SEM; Student’s *t*-test). **D** The expression of P0 protein in Schwann cells transfected with siRNA-MOGS (si-MOGS-3) and siRNA control (si-Ctr). *p-value < 0.05 versus si-Ctr (*n* = 3, mean ± SEM; Student’s *t*-test)

Given that MOGS and many MOGS-associated metabolism-related genes were differentially expressed in sciatic nerves after peripheral nerve injury, we hypothesized that these dysregulated metabolism-related genes may regulate the behavior of Schwann cells in the nerve segments and participate in the regulation of nerve regeneration. Schwann cells were transfected with siRNA against MOGS to examine the direct effect of MOGS knockdown on Schwann cell phenotype. RT-PCR showed that si-MOGS-3 led to significant reduction in the mRNA expression of MOGS, and thus, this siRNA was used for subsequent functional investigations (Fig. 3A).

The effect of MOGS on Schwann cell differentiation was also determined. Schwann cells were cultured in control medium (Dulbecco’s modified Eagle’s medium nutrient mixture F12 (DMEM/F12) with 0.5% fetal bovine serum (FBS)) or differentiation medium (DMEM/F12, 0.5% FBS, 20 ng/ml heregulin (HRG) and 1 mM db-cAMP) prior to real-time RT-PCR experiments. Compared with cells cultured in the control medium, Schwann cells cultured in the differentiation medium expressed higher level of myelin gene *P0* (also known as Mpz, P0-positive cells differentiated specifically into mature Schwann cells) as well as decreased level of *p75* (p75-positive cells had multidirectional differentiation potential) (Fig. 3B). Subsequently, Schwann cells transfected with MOGS-siRNA or negative control were cultured in the differentiation medium to examine the effect of MOGS on Schwann cell differentiation. Compared with cells transfected with negative control, Schwann cells transfected with MOGS-siRNA had a slightly higher (but not significant) mRNA level of *P0* and significantly lower expression of *p75* (Fig. 3C). At the protein level, *P0* expression was significantly higher in Schwann cells transfected with MOGS-siRNA than in those transfected with negative control (Fig. 3D). These observations suggested that silencing MOGS promoted the differentiation of Schwann cells.

### MOGS Negatively Affected Schwann Cell Proliferation

**Fig. 4:**
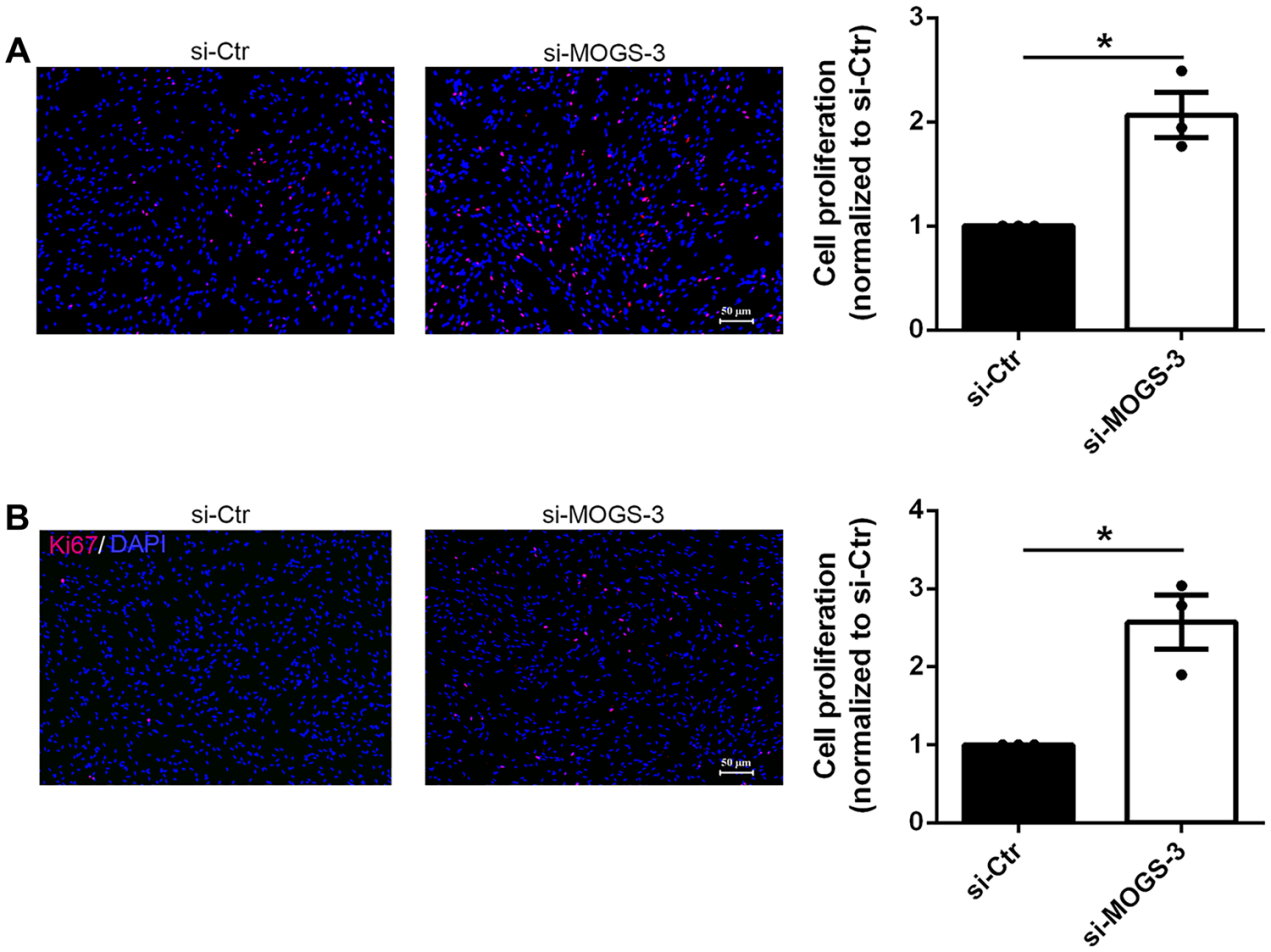
Knockdown of MOGS promotes Schwann cell proliferation. **A** Transfection of Schwann cells with si-MOGS increased EdU incorporation. **B** Transfection of Schwann cells with si-MOGS increased the immunostaining signals of Ki67. *p-value < 0.05 versus si-Ctr (*n* = 3, mean ± SEM; Student’s *t*-test). Scale bars represent 50 μm

After MOGS knockdown, Schwann cells were exposed to EdU to determine whether MOGS knockdown would influence DNA synthesis of Schwann cells. In the EdU incorporation assay, EdU-positive proliferating cells were marked in red, and the nuclei were marked in blue. Representative images of EdU incorporation assay showed a significant increase in the number of EdU-positive cells after MOGS transfection, while the number of total cells was similar (Fig. 4A). The proliferation rate was calculated by dividing the number of EdU-positive cells by the number of total cells. Data showed that after si-MOGS-3 transfection, the proliferation rate of Schwann cells was increased by about twofold compared to the proliferation rate of Schwann cells transfected with a control siRNA (si-Ctr) (Fig. 4A).

The proliferation status of Schwann cells was also examined by the quantification of proliferation protein Ki67. Ki67 is typically expressed in cells in G1, S, and G2/M phases, but absent in non-proliferation G0 cells. Immunostaining images showed that the number of Ki67-positive cells was significantly increased after transfection with si-MOGS-3 (Fig. 4B). These observations indicated that MOGS could suppress Schwann cell proliferation.

### MOGS Negatively Affected Schwann Cell Migration

**Fig. 5:**
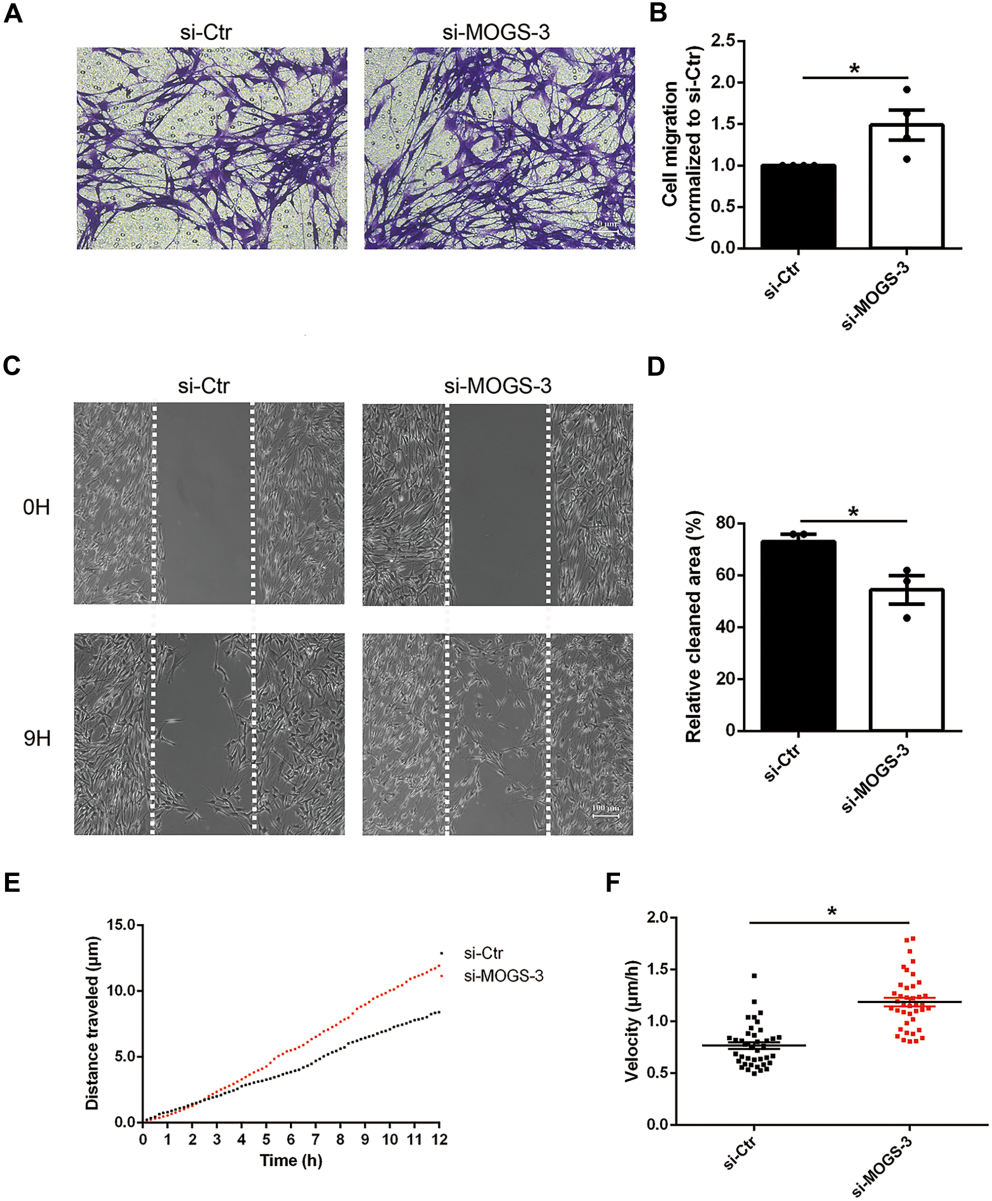
Knockdown of MOGS promotes Schwann cell migration. **A** Representative images and **B** summarized proliferation rates of Schwann cells transfected with si-Ctr and si-MOGS-3 in Transwell migration assay. Violet color indicates cells migrated through transwell chambers. *p-value < 0.05 versus si-Ctr (*n* = 3, mean ± SEM; Student’s *t* test). Scale bars represent 50 μm. **C** Representative images and **D** summarized relative clear areas of Schwann cells transfected with si-Ctr and si-MOGS-3 in wound healing assay. Scale bars represent 100 μm. *p-value < 0.05 versus si-Ctr (*n* = 3, mean ± SEM; Student’s *t*-test). **E** Transfection of Schwann cells with si-MOGS-3 increased migration distance and velocity. **F** Transfection of Schwann cells with si-MOGS-3 increased migration velocity. *p-value < 0.05 versus si-Ctr (data were displayed in a scatter plot from four experiments using ten replicate wells, mean ± SEM; Student’s *t*-test)

The influence of MOGS on the migration of Schwann cells was investigated using a Transwell migration assay. In all groups, Schwann cells migrated from the upper chamber of the Transwell toward the bottom chamber. Numerous crystal violet–stained cells were detected in the bottom chambers of Transwell with Schwann cells transfected with si-MOGS-3 (Fig. 5A). Migrated cells were dissolved in 33% acetic acid and the absorbance of crystal violet staining was measured to quantify changes of cell migration. The absorbance reading of Schwann cells transfected with si-MOGS was higher than the reading of cells transfected with si-Ctr, indicating that MOGS knockdown promotes Schwann cell migration (Fig. 5B).

Transfected Schwann cells were further subjected to a wound healing assay. An equal wide wound area was generated at 0 h after wound healing culture insert removal. However, after 9 h culture, the blank space was obviously smaller in Schwann cells transfected with si-MOGS (Fig. 5C). Measurement of the remaining clear area showed that in Schwann cells transfected with si-Ctr, less than 30% of the wound area was filled with migrated cells. In contrast, in Schwann cells transfected with si-MOGS-3, about 45% of wound area was occupied with migrated cells (Fig. 5D).

Live cell imaging was used to monitor and determine the migration trajectory and distance of siRNA-transfected Schwann cells over time. Migration distance of Schwann cells transfected with si-MOGS was lower than cells transfected with si-Ctr (Fig. 5E). The mean velocity calculated by ImageJ showed that the migration velocity of Schwann cells transfected with si-MOGS-3 was higher than Schwann cells transfected with si-Ctr (Fig. 5F).

## Discussion

Schwann cells undergo dedifferentiation, proliferation, and migration after peripheral nerve injury. The adaptive reprogramming of Schwann cells contributes to the construction of a regenerative permissive microenvironment and benefits tissue remodeling (Jessen and Arthur-Farraj 2019; Nocera and Jacob 2020). Many factors that modulate the phenotype of Schwann cells have been demonstrated to be essential regulators of peripheral nerve regeneration. For instance, growth factor betacellulin stimulates the migration of Schwann cells and the elongation of neurites in vitro and facilitates the formation of Schwann cell–formed cord in the nerve bridge and axon growth in vivo (Wang et al. 2021). In contrast, elevation of *LIF*, a gene coding for leukemia inhibitory factor, hinders Schwann cell proliferation and migration while knockdown of *LIF* facilitates the morphological and functional recovery of peripheral nerve injury (Chen et al. 2021).

In this study, we examined the biological functions of MOGS using siRNA-mediated silencing and found that reduced mRNA expression of MOGS stimulated Schwann cell differentiation, proliferation, and migration. These observations implied that repressed MOGS expression induced phenotype modulation, high proliferation, and migration of Schwann cells. In addition, knockdown of MOGS in Schwann cells lowered the expression of *FASN* (Fig. S1). Therefore, genetic modification of Schwann cells with MOGS may influence surrounding neurons through a non-cell-autonomous mechanism and affect axon growth following peripheral nerve injury.

Given that MOGS was found to be upregulated in sciatic nerves after peripheral nerve injury, elevated MOGS might play an inhibitory role on Schwann cell reprogramming. Similar to these observations, our recent study showed that miR-29a-3p, a non-coding miRNA that was upregulated in sciatic nerves after nerve injury, suppressed Schwann cell proliferation and migration and hampered the recovery of both motor and sensory functions (Shen et al. 2021). Hence, strategies to reduce the expression of MOGS may improve peripheral nerve regeneration and could be applied to treat peripheral nerve injury.

Non-coding RNAs, for instance, can modulate the expression of target genes and can be utilized as therapeutic strategies (Yu et al. 2015). Using bio-prediction tools, TargetScan, miRWalk, miRcode, and RNA22, miR-140-5p, miR-449c-5p, and miR-761 were screened and selected as candidate regulatory miRNAs of MOGS. Expression profiles of mouse sciatic nerve distal segments post-injury showed that miR-140 is an injury-regulated miRNA whose overexpression suppresses the differentiation and remyelination of Schwann cells (Viader et al. 2011). In vivo observations showed that transplantation of miR-449-transfected adipose-derived stem cells benefits facial nerve regeneration (Tan et al. 2019). Although the direct function of miR-761 in Schwann cells remains unknown, a recent study showed that transfection of neurons with miR-761 increases neurite length (Wang et al. 2020). The beneficial roles of miR-140, miR-449, and miR-761, together with the promoting role of MOGS siRNA on Schwann cell plasticity, imply that these candidate miRNAs may regulate the peripheral nerve repair process via targeting and suppressing MOGS expression.

Identification of genes that are closely associated with MOGS and functional annotations of these genes emphasizes the significant involvement of cellular metabolism. Moreover, our previous study of transcription factors showed that *TFAP2A*, a gene coding for transcription factor activating enhancer binding protein 2 alpha, exhibited similar elevated expression patterns as MOGS (Zhang et al. 2019). TFAP2A is reported to influence the biogenesis of lipid droplets (Scott et al. 2018). Given the involvement of TFAP2A in energy supply and metabolism, we hypothesized that TFAP2A transcription factor may target MOGS and thus participate in the regulation of Schwann cells. The binding region of TFAP2A and the promoter region of MOGS were analyzed, and three possible binding site sequences were found (Fig. S2). The outcomes jointly reflect that TFAP2A may directly bind to MOGS, positively regulate the transcription of MOGS, and modulate the behaviors of Schwann cells and neurons.

Collectively, this study revealed elevated expression patterns of MOGS in sciatic nerves after peripheral nerve injury, identified associated molecules of MOGS and the functional effects of MOGS on Schwann cell phenotype modulation, and thus improved the understanding of the importance of metabolism-related genes in peripheral nerve regeneration.

## Supplementary Information

Below is the link to the electronic supplementary material.

Supplementary file1 (TIF 56 kb). Fig. S1. Effect of MOGS siRNA on FASN expression in Schwann cells. RT-PCR quantification of the relative mRNA expression levels of FASN after transfection of Schwann cells with si-Ctr and si-MOGS-3. The mRNA levels of FASN were higher in si-Ctr transfected Schwann cells than siRNA-MOGS transfected Schwann cells. *p-value < 0.05 versus si-Ctr (n = 3, mean ± SEM; Student’s t-test)

Supplementary file2 (TIF 3954 kb). Fig. S2. Prediction of TFAP2A binding sites in the MOGS promoter region using bioinformatics methods. (A) The binding motif of TFAP2A. (B) The three possible binding site sequences -1575 to -1561, -81 to -73 and -64 to -79 were identified from the JASPAR database (https://ngdc.cncb.ac.cn/databasecommons/database/id/176) and AnimalTFDB v3.0 (http://bioinfo.life.hust.edu.cn/AnimalTFDB/#!/citation) in the promoter region of MOGS gene

## Data Availability

Data are available from the corresponding authors upon reasonable request.

## Abbreviations

MOGS: Mannosyl-oligosaccharide glucosidase
FASN: Fatty acid synthase
DDOST: Dolichyl-diphosphooligosaccharide protein glycosyltransferase non-catalytic subunit
DAD1: Defender against cell death 1
TUSC3: Tumor suppressor candidate 3
ISOC1: Isochorismatase domain containing 1
ISOC2: Isochorismatase domain containing 2
LRRK2: Leucine-rich repeat kinase 2
YBX1: Y-Box binding protein 1
TIMM44: Translocase of inner mitochondrial membrane 44
STT3A: STT3 oligosaccharyltransferase complex catalytic subunit A
STT3B: STT3 oligosaccharyltransferase complex catalytic subunit B
RPN1: Ribophorin I
MAGT1: Magnesium transporter 1
GANAB: Glucosidase II alpha subunit
GANC: Glucosidase alpha neutral C
