# Supplementary figures and images for: Metabolism-related MOGS Gene is Dysregulated After Peripheral Nerve Injury and Negatively Regulates Schwann Cell Plasticity
Source: J Mol Neurosci. 2022 May 16;72(6):1402–12. doi: 10.1007/s12031-022-02024-8 (PMC9170655; doi:10.1007/s12031-022-02024-8)

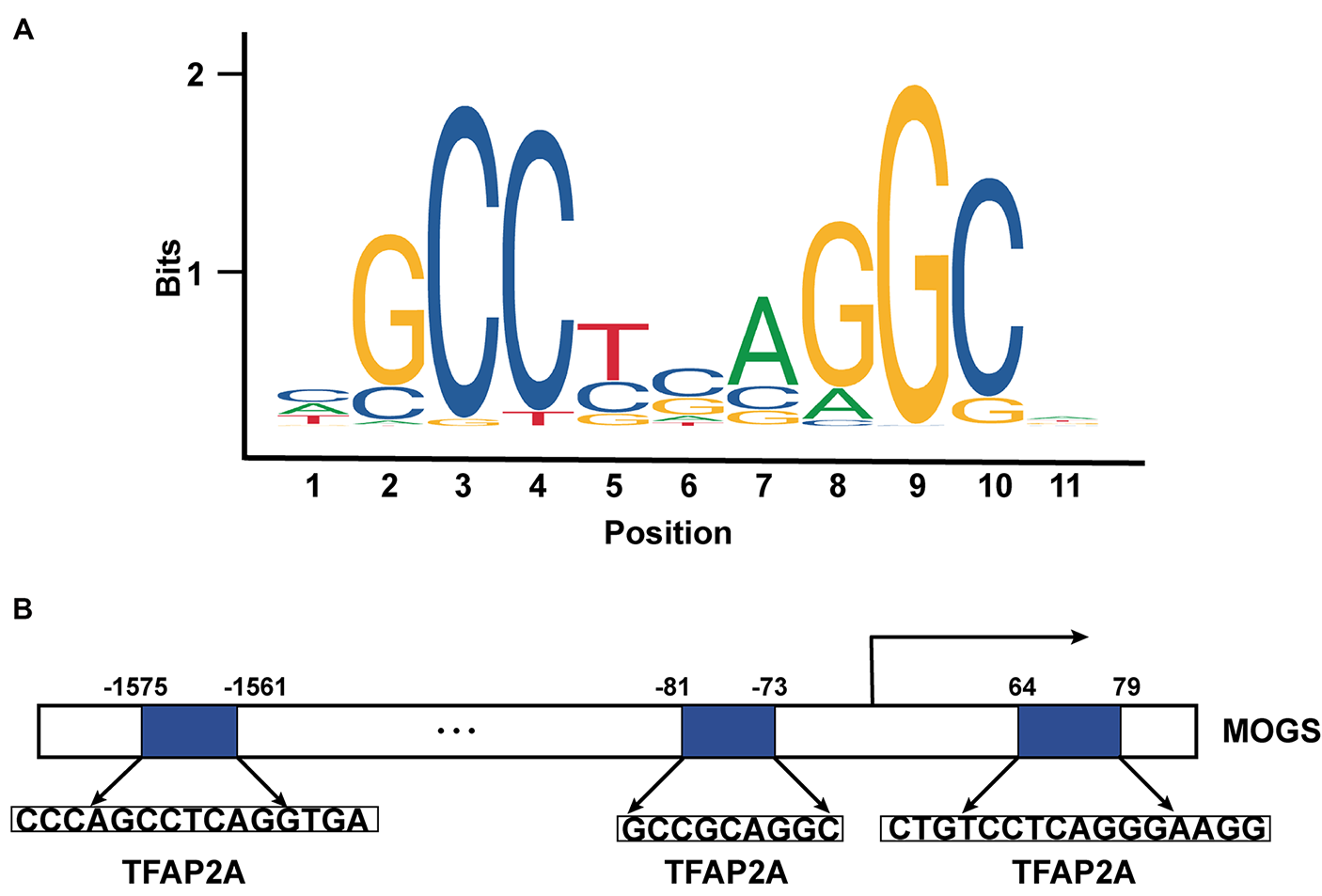

Supplement: Supplementary file 2 — Supplementary file2 (TIF 3954 kb). Fig. S2. Prediction of TFAP2A binding sites in the MOGS promoter region using bioinformatics methods. (A) The binding motif of TFAP2A. (B) The three possible binding site sequences -1575 to -1561, -81 to -73 and -64 to -79 were identified from the JASPAR database (https://ngdc.cncb.ac.cn/databasecommons/database/id/176) and AnimalTFDB v3.0 (http://bioinfo.life.hust.edu.cn/AnimalTFDB/#!/citation) in the promoter region of MOGS gene [file 12031_2022_2024_MOESM2_ESM.tif]
